# Multiplexed CRISPR-Cas9 system in a single adeno-associated virus to simultaneously knock out redundant clock genes

**DOI:** 10.1038/s41598-021-82287-0

**Published:** 2021-01-28

**Authors:** Boil Kim, Jihoon Kim, Minjeong Chun, Inah Park, Damhyeon Kwak, Mijung Choi, Kyungjin Kim, Han Kyoung Choe

**Affiliations:** 1 Department of Brain and Cognitive Sciences, Daegu Gyeongbuk Institute of Science and Technology (DGIST), E4-311, 333 Technojoongang-daero, Dalseong-gun, Daegu, 42988 South Korea; 2 Convergence Research Advanced Centre for Olfaction, Daegu Gyeongbuk Institute of Science and Technology (DGIST), Daegu, South Korea; 3 Korean Brain Research Institute (KBRI), Daegu, South Korea

**Keywords:** Molecular biology, Neuroscience

## Abstract

The mammalian molecular clock is based on a transcription-translation feedback loop (TTFL) comprising the Period1, 2 (*Per1, 2*), Cryptochrome1, 2 (*Cry1, 2*), and Brain and Muscle ARNT-Like 1 (*Bmal1*) genes. The robustness of the TTFL is attributed to genetic redundancy among some essential clock genes, deterring genetic studies on molecular clocks using genome editing targeting single genes. To manipulate multiple clock genes in a streamlined and efficient manner, we developed a CRISPR-Cas9-based single adeno-associated viral (AAV) system targeting the circadian clock (CSAC) for essential clock genes including *Per*s, *Cry*s, or *Bmal1*. First, we tested several single guide RNAs (sgRNAs) targeting individual clock genes in silico and validated their efficiency in Neuro2a cells. To target multiple genes, multiplex sgRNA plasmids were constructed using Golden Gate assembly and packaged into AAVs. CSAC efficiency was evident through protein downregulation in vitro and ablated molecular oscillation ex vivo. We also measured the efficiency of CSAC in vivo by assessing circadian rhythms after injecting CSAC into the suprachiasmatic nuclei of Cas9-expressing knock-in mice. Circadian locomotor activity and body temperature rhythms were severely disrupted in these mice, indicating that our CSAC is a simple yet powerful tool for investigating the molecular clock in vivo.

## Introduction

The circadian clock regulates most physiological processes. In mammals, this system has a hierarchical structure with a master clock machinery located in the suprachiasmatic nucleus (SCN) that synchronizes local clocks in almost every organ, tissue, and cell via humoral and neural signaling^1,2^. All individual cellular circadian oscillators, master or local, are driven by molecular clockwork operating via transcription-translation negative feedback loops (TTFLs), which are primarily regulated by a transcriptional feedback loop comprising positive regulators *Bmal1* and *Clock* and negative regulators Cryptochrome (*Cry*) and Period (*Per*), which are finely modulated by post-transcriptional and -translational modifications^3,4^. Defects in local molecular oscillators can cause the abnormal functioning of the corresponding tissues or organs, resulting in sleep disorders, mood disorders, obesity, and autoimmunity^5^. Although recent advancements in single-cell RNA sequencing have advanced our understanding of systemic organization of diverse cell types in the body, the mechanism underlying the physiological role of the circadian clock in these diverse cell types remain unclear owing to the lack of cost- and labor-efficient techniques^6,7^. The expanding repertoire of *Cre* driver lines could serve as a molecular handle to manipulate clock genes in various cell types^8,9^; however, an efficient and approachable technique to knock out molecular clocks in specific cell types is also required to facilitate our understanding of the role of local clocks.

Genetically engineered mice help address the roles of local molecular clocks and the mechanisms underlying their physiological and pathological effects. *Mus musculus* possesses two functional *Period* genes (*Per1* and *Per2*) whose double knockout (KO) leads to immediate and complete arrhythmicity, whereas single *Per1* or *Per2* KO animals display compromised rhythmicity, including altered behavioral periods, delayed arrhythmicity, or residual ultradian behavioral patterns^10–13^. Similarly, the double KO of both *Cry1* and *Cry2* is required for complete arrhythmicity^14,15^, since single *Cry1* or *Cry2* KO only affects period length (one shortening and the other lengthening the period) while preserving oscillation^15,16^. *Bmal1* is an essential core clock component whose single KO causes immediate and complete arrhythmicity^17^. Although multiple animal models have displayed circadian phenotypes, they differ in their non-circadian phenotypes. For instance, muscular and skeletal degeneration are only observed in *Bmal1* KO^17^. Both *Per* and *Bmal1* KO affected mood regulation. *Per1,2*^-/-^ mice displaying increased anxiety without hyperactivity^18^, while shRNA-mediated *Bmal1* knockdown in the SCN led to both increased anxiety and depression^19^. Furthermore, *Clock* delta19 mutants display low anxiety and depression in a circadian-dependent manner^20^, while *Rev-erbα* KO mice display decreased anxiety and depression^21^. These conflicting findings highlight the importance for an efficient genome editing tool to investigate local clocks in relevant tissues.

The CRISPR-Cas9 system is derived from the adaptive immune system of prokaryotes and cleaves specific sequences in foreign DNA^22^. For genome editing, guide RNA (gRNA) containing a 20-bp spacer sequence followed by PAM 5′-NGG enables the Cas9-gRNA complex to recognize and be recruited to genomic loci complementary to the spacer sequence. Cas9 then generates a double-strand break (DSB) that is rectified through a DNA repair system, such as the non-homologous end joining pathway, thus generating a quasi-random indel at the DSB to potentially produce frame-shift mutation^22^. In circadian genes, several applications of the CRISPR-Cas9 system have been reported for robust and efficient knockout of molecular clock components. Korge et al*.* first used CRISPR-Cas9-mediated gene targeting in chronobiology studies to generate *FBXL3* KO U2-OS cells^23^ and have recently extended this application to generate *CRY1* and *CRY2* KO U2-OS cells. Furthermore, the CRISPR-Cas9 system has been used to generate KO animals lacking core clock genes in mice, macaques, monarch butterflies, and *Neurospora crassa*^24–27^. Virally delivered single guide RNAs (sgRNAs) targeting core clock components are reportedly efficient in vitro and in vivo^28,29^; however, CRISPR-Cas9 system has only been applied for chronobiological analysis of single genes at a time, thus limiting its ability to study redundant clock genes.

In this study, we developed a CRISPR-Cas9-based single adeno-associated viral (AAV) system targeting the circadian clock (CSAC), using CRISPR-Cas9-mediated genome editing techniques and an AAV vector system to simultaneously knockout multiple components of the core clock machinery. We assembled a set of individual sgRNAs into a single AAV and demonstrated its multiplexing efficiency in vitro and in vivo. We believe our system would greatly facilitate further studies on the molecular clock in diverse cell types because AAV can efficiently infect numerous different cell types in vitro and in vivo.

## Results

### Design and evaluation of sgRNAs targeting core clock gene family members

**Figure 1:**
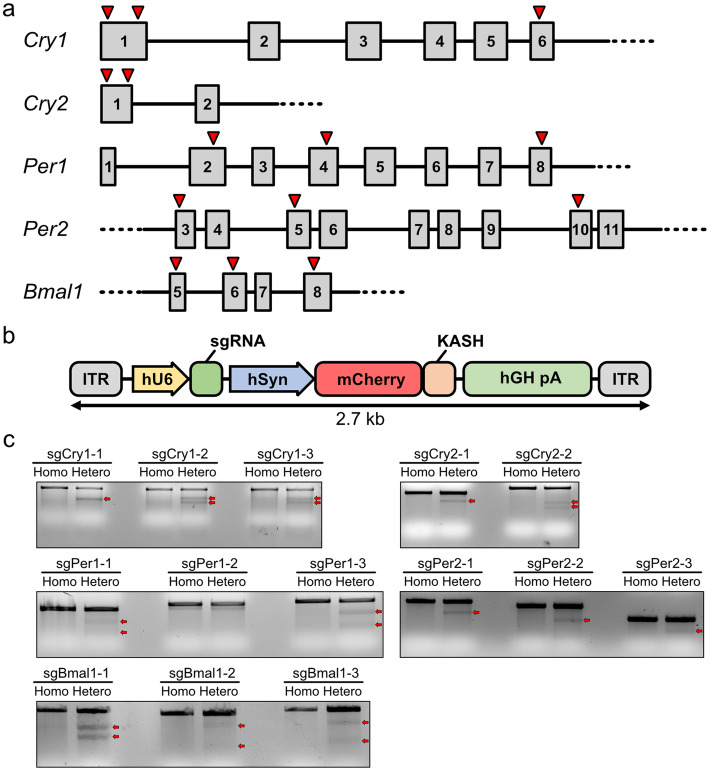
Design and evaluation of single guide RNAs (sgRNAs) targeting core molecular clock family members. (**a**) Genomic location of sgRNAs targeting *Cry1*, *Cry2*, *Per1*, *Per2*, and *Bmal1*. Gray boxes indicate exons. Lines indicate introns. Red arrows indicate the location of sgRNAs. (**b**) Structure of sgRNA expression vectors driven by the U6 promoter (hU6). ITR, inverted terminal repeat; hSyn, human synapsin promoter; KASH, Klarsicht ANC-1 syne homology domain for nuclear membrane targeting; hGH pA, poly adenylation signal derived from human growth hormone. (**c**) Mutation efficiency determined by Surveyor cleavage assays. Red arrows indicate cleaved PCR products, indicating introduced mutations. Homo, homoduplex of PCR-amplified DNA fragments from the genomic DNA of non-transfected Neuro2a cells; Hetero, heteroduplex of PCR-amplified DNA fragments from the genomic DNA of non-transfected and sgRNA-expressing-plasmid-transfected Neuro2a cells.

To obtain a set of single guide RNAs (sgRNAs) capable of knocking out essential circadian clock genes, we designed sgRNAs targeting core clock genes (*Cry1*, *Cry2*, *Per1*, *Per2*, and *Bmal1*) using CHOPCHOP, a web-implemented target-site selecting algorithm for CRISPR-Cas9^30^. Among the predicted sgRNAs, we selected three, which targeted early exons to maximize the effect of the frame shift-mediated non-sense mutation (Fig. 1a). The individual sgRNAs were then cloned into a U6 promoter-driven sgRNA expression vector to evaluate their mutation-inducing efficiency (Fig. 1b). Each sgRNA-expressing plasmid was transfected into the Neuro2a neuroblastoma cell line with *SpCas9*-expressing plasmids. The mutation rate of the harvested genomic DNA was evaluated using Surveyor nuclease mismatch cleavage assay, which can detect potential mutations in the genomic DNA of transfected cells, be it a single base conversion or an indel mutation.

All the genomic DNA of Neuro2a cells transfected with plasmids expressing sgRNA targeting *Cry1* (sgCry1) yielded cleaved DNA bands in the mutation mismatch cleavage assay (Fig. 1c, arrows). In the sgCry2 group, both sgRNAs caused cleavage, but not as abundantly as those in the sgCry1 groups. Since a single AAV can harbor three sgRNA-expressing cassettes at maximum, we combined one sgRNA targeting *Cry1* with high mutagenic potential (sgCry1-2) and two sgRNAs targeting *Cry2* with modest mutagenic potential (sgCry2-1 and sgCry2-2) to increase the probability of simultaneous KO of *Cry1* and *Cry2* (Fig. 1c). sgRNAs for *Pers* were similarly selected. Since the sgPer1-3, sgPer2-1, and sgPer2-2 groups produced intense cleavage bands, these three sgRNA targeting *Per*s were selected for the multiplexed AAV vector. For *Bmal1*, we combined two well-functioning sgRNAs targeting different *Bmal1* sites (sgBmal1-1 and sgBmal1-3) to increase the efficacy of *Bmal1* KO. The sgRNA sequences are shown in Table 1.

**Table 1:** Nucleotide sequences of sgRNAs selected for CSAC construction.

sgRNA	sgRNA sequence (5′ → 3′)	Strand	Exon	Distance from ATG (bp)
sgCry1-2	GGTCGAGGATATAGACGCAGCGG	+	1	95
sgCry2-1	CTGTGGGCATCAACCGATGGAGG	−	1	210
sgCry2-2	TCGGTTGATGCCCACAGACGAGG	+	1	187
sgPer1-3	GGATGCGCTCGGCAATGAGTAGG	−	8	1013
sgPer2-1	GGATGGCGAGCATCAAGGGCCGG	+	10	1118
sgPer2-2	GAGCACAACCCCTCCACGAGCGG	−	3	278
sgBmal1-1	GGAACCGGAGAGTAGGTCGGTGG	−	5	78
sgBmal1-3	GCCTCTTTTCAATCTGACTGTGG	−	8	251

Underlined sequences indicated PAM sequence. Strand denotes whether sgRNA recognizes sense (+) or anti-sense (−) strand of target gene.

**Figure 2:**
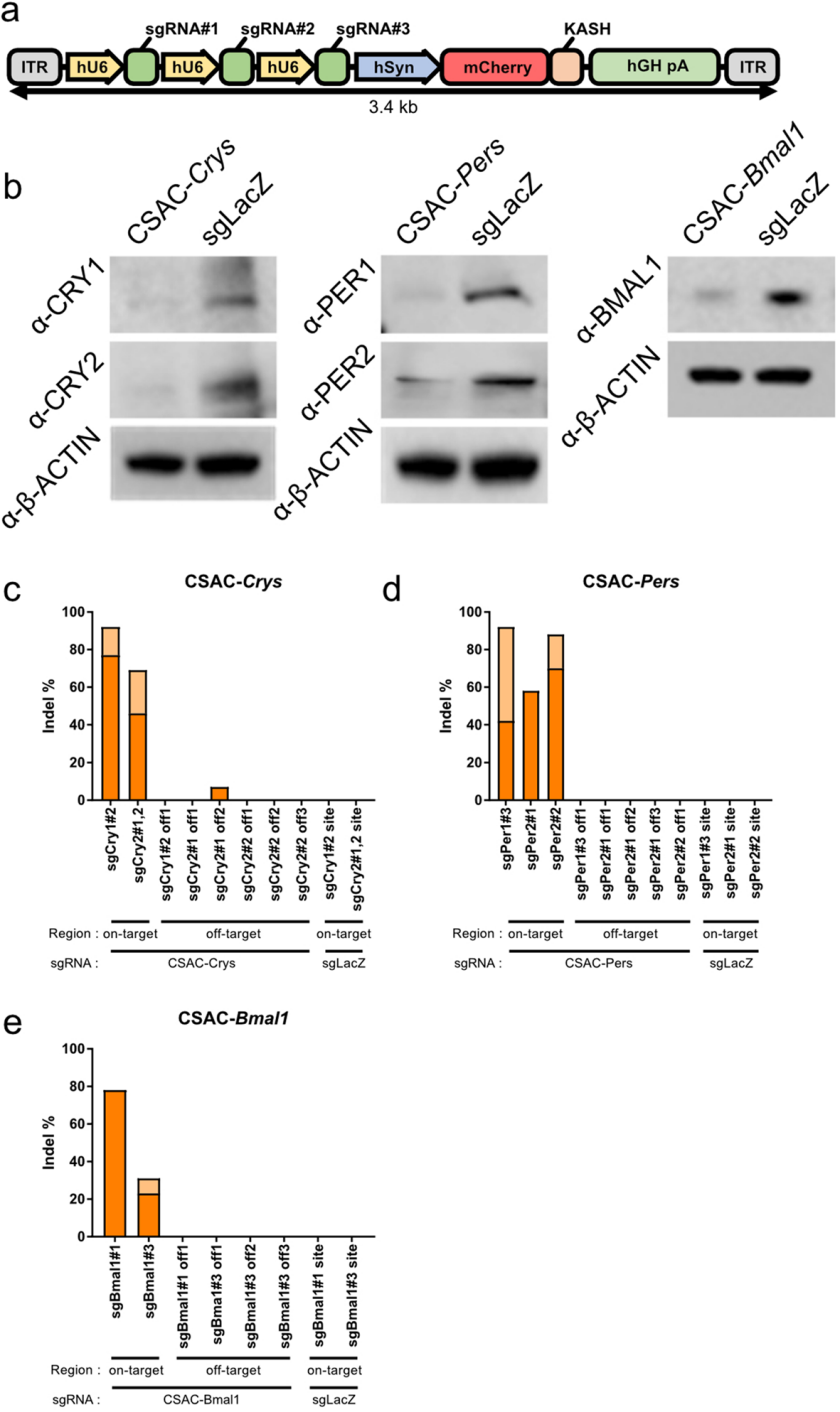
Reduced protein levels of core clock components by genome editing with a multiplexed sgRNA-expressing cassette. (**a**) Structure of multiplexed U6 promoter-driven sgRNA expression cassettes for AAV packaging. (**b**) Representative western blots of protein extracts from Neuro2a-Cas9 cells transfected with CSAC- *Crys* (left), CSAC-*Pers* (middle), or CSAC-*Bmal1* (right). Proteins from CSAC-*Crys*-infected cells were probed with anti-CRY1 and anti-CRY2 antibodies. Proteins from CSAC-*Pers*-infected cells were probed with anti-PER1 and anti-PER2 antibodies. Proteins from CSAC-*Bmal1*-infected cells were probed with anti-Bmal1 antibodies, with anti-β-actin antibodies used as an internal control for total protein abundance. (**c**–**e**) Percentage of indel mutation induced by CSAC-*Crys* (**c**), CSAC-*Pers* (**d**), CSAC-*Bmal1* (**e**), and LacZ, respectively. On-target and off-target sites of each sgRNA included in CSAC are indicated. Orange bar: frame-shift mutation; Light orange bar: non-frame-shift mutation.

Thereafter, we examined the genome editing efficacy of the multiplexed sgRNAs in downregulating protein expression. Neuro2a cells stably expressing Cas9:EGFP (hereafter referred to as Neuro2a-Cas9 cells) were transfected with plasmids harboring multiplexed U6-mediated sgRNA expression cassettes (Fig. 2a) before protein extracts were isolated and subjected to western blot assays with antibodies against CRYs, PERs, or BMAL1. CRY1 and CRY2 protein levels were markedly lower in Neuro2a-Cas9 cells transfected with plasmids expressing sgCrys than in those transfected with the sgLacZ control (Fig. 2b; Fig. S1). Similarly, the abundance of PER1 and PER2 protein in the sgPers-transfected group was less than half of that in the sgLacZ control group, while the sgBmal1s downregulated BMAL1. Accordingly, we named the multiplexed sgRNA-expressing AAV vector system “CSAC”.

Furthermore, we analyzed CSAC-induced mutations at single-nucleotide resolution through Sanger sequencing. We transfected Neuro2a cells with Cas9-expressing plasmid and CSAC-expressing plasmid, sorted the cells on the basis of Cas9 and sgRNA expression levels, and sequenced the on-target and predicted off-target site of sgRNAs (Table 2) included in CSAC in FACS-sorted Neuro2A cells. CSAC-induced mutagenesis resulted in various mutations ranging from deletions, insertions, and base substitutions (Fig. S2). To assess the knockout efficiency of CSAC, we determined the percentage of indel mutations (Fig. 2c–e), particularly focusing on frame-shift mutations. Indeed, the indel mutation leading to frame-shift mutation was predominant in CSAC-Crys-, CSAC-Pers-, and CSAC-Bmal1-transfected cells. Together, mutation assays of genomic DNA from transfected cells and western blot assays indicated successful targeted gene KO.

**Table 2:** Nucleotide sequences of predicted off-target sites of sgRNAs selected for CSAC.

Off-target	Sequence (5′ → 3′)	Strand	Location
sgBmal1#1 off-target1	GGAACTGGAGAGTACGTGGGAGG	−	chr12:60,954,060
sgBmal1#3 off-target1	GCCACATTTCAATATGACTGGGG	+	chr1:54,158,776
sgBmal1#3 off-target2	GTCTCTTTTCAAGCAGACTGAGG	+	chr13:34,815,040
sgBmal1#3 off-target3	GCCACTTTCCAATCAGACTGGGG	+	chr13:96,050,061
sgCry1#2 off-target1	CCTCGAGGATATAGACACAGGGG	−	chr6:67,957,767
sgCry2#1 off-target1	CTGTGGGCGACAACCGATTGGGG	+	chr10:69,600,590
sgCry2#1 off-target2	CTGTAGGCCTCAACCAATGGGGG	−	chr2:34,633,756
sgCry2#2 off-target1	TCCTTTGATGCCCACAGATGGGG	−	chr11:8,020,808
sgCry2#2 off-target2	TCGGTGGATGCCAACAGACTTGG	+	chr2:129,503,000
sgCry2#2 off-target3	TCTCTTGATGCTCACAGACGTGG	+	chr5:48,736,471
sgPer1#3 off-target1	GGAGGCCCTCGGCAATGAGAGGG	+	chr12:33,074,634
sgPer2#1 off-target1	GGATGGGGATCCTCAAGGGCAGG	+	chr11:69,548,074
sgPer2#1 off-target2	GGATGGAGACCATCAAGGCCAGG	+	chr11:113,289,426
sgPer2#1 off-target3	GGGTGGCAAACATCAAGGGCAGG	−	chr17:30,945,601
sgPer2#2 off-target1	GAGAACAACCCCTCCACCATAGG	−	chr5:36,706,704

Underlined sequences indicated PAM sequence. Strand denotes whether sgRNA recognizes sense (+) or anti-sense (−) strand of target gene. Red characters indicate mismatch to on-target sequence.

### CSAC dampened molecular clock oscillation in SCN slice cultures

**Figure 3:**
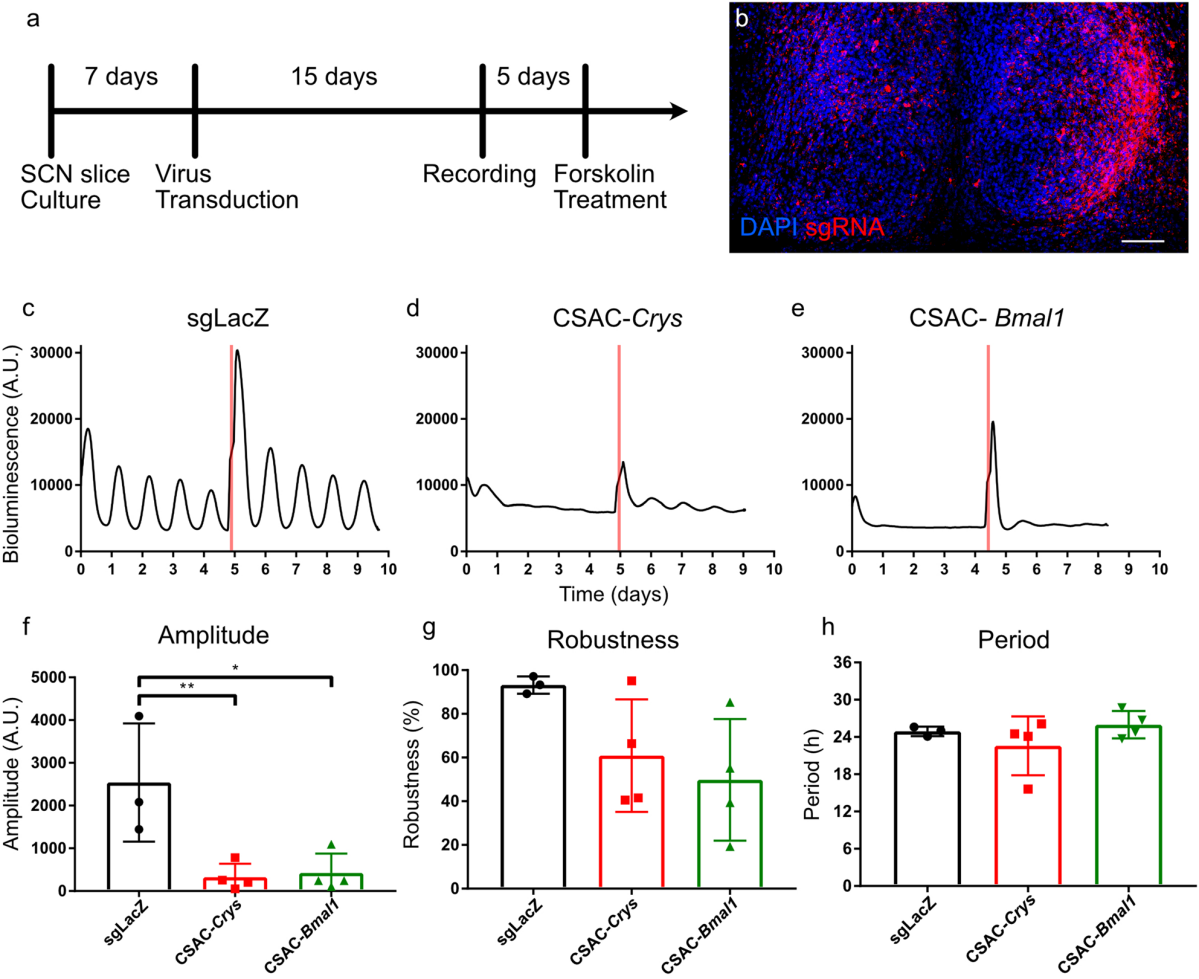
CSAC obliterated molecular clock oscillation ex vivo. (**a**) Timeline of organotypic slice culture preparation and viral infection. (**b**) Representative confocal microscopy image of organotypically cultured SCN from mPER2::LUC;Cas9 mice. Scale bar: 100 μm. (**c**–**e**) Representative profiles of PER2::LUC mice with CSAC. Bioluminescence signals from SCN explant cultures infected with either sgLacZ (**c**), CSAC-*Crys* (**d**), or CSAC-*Bmal1* (**e**) were continuously monitored. Red lines indicate forskolin treatment. (**f**–**h**) Oscillation amplitude (**f**), robustness (**g**), and period (**h**) in PER2::LUC slice cultures infected with AAV analyzed using Cosinor. * *p* < 0.05; ** *p* < 0.01 by Tukey’s post-hoc test (*n* = 3–4 slices per group).

To determine whether CSAC-mediated protein downregulation mitigated central molecular clock oscillation at a population level, we monitored the oscillation of bioluminescence emitted from organotyptic SCN slice cultures from neonatal knock-in animals expressing a PER2 and LUCIFERASE fusion protein and constitutively expressing Cas9 (PER2::LUC;Cas9; Fig. 3a)^31,32^. Bath application of CSAC, followed by 2-week incubation, enabled the widespread expression of fluorescent reporter proteins, thus highlighting sgRNA expression in the SCN (Fig. 3b). The SCN explants expressing control sgLacZ exhibited robust circadian oscillation in PER2 expression during the recording session (Fig. 3c); however, PER2 oscillation was severely dampened in SCN cultures infected with either CSAC-*Cry*s or CSAC-*Bmal1* after the first peak induced by medium change, without a noticeable increase in PER2::LUC expression (Fig. 3d,e). To rule out the possibility that CSAC damages SCN cells or interferes with genes regulating PER2, we pharmacologically activated PER2 by administering forskolin, an adenylyl cyclase activator^33^. In all groups, forskolin treatment robustly induced PER2::LUC expression, regardless of CSAC. Furthermore, CSAC induced dampening of the molecular clock, with CSAC-*Crys* and CSAC-*Bmal1* both significantly suppressing the amplitude of PER2::LUC oscillation (Fig. 3f). Moreover, CSAC affected the robustness of bioluminescence oscillation, although to a lesser degree than its impact on amplitude owing to the succession of suppressed peaks (Fig. 3g) and did not significantly affect the period of residual molecular oscillation (Fig. 3h). Together, these findings indicate that CSAC effectively and specifically dampens molecular clock oscillation ex vivo.

### SCN-injected CSAC induced arrhythmicity in locomotor activity and body temperature

**Figure 4:**
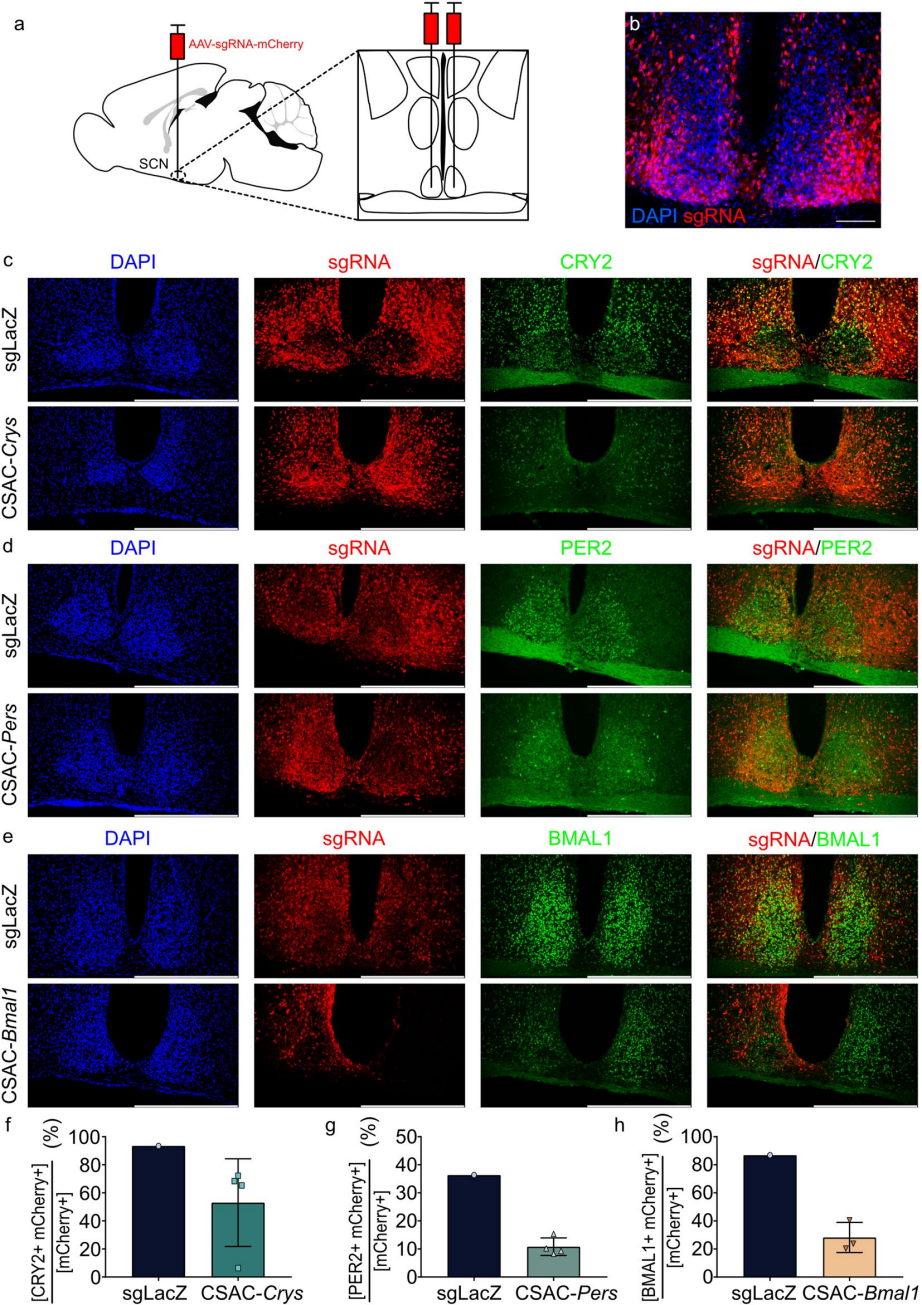
SCN injection with CSAC. (**a**) Schematic representation of stereotaxic CSAC injection into the SCN. AAVs expressing sgRNAs targeting core clock components and the mCherry fluorescent marker were bilaterally injected into the SCN of Cas9-expressing mice. (**b**) Representative confocal microscopic image of the brain section containing CSAC-injected SCN. sgRNA-expressing cells are co-labeled with mCherry as a surrogate marker. Scale bar = 100 μm. (**c**–**e**) Immunohistochemical analysis of brain sections containing the SCN of mice injected with CSAC-*Crys* (**c**), CSAC-*Pers* (**d**), and CSAC-*Bmal1* (**e**). Scale bar: 100 μm for (**b**) and 500 μm for (**c**–**e**). (**f**–**h**) Co-expression ratio of targeted clock proteins and CSAC. Percentage of clock protein-positive and mCherry-positive cells over mCherry-positive cells were quantified. (**f**) Co-expression percentages of CRY2 and mCherry are indicated for the sgLacZ or CSAC-*Crys* group. (**g**) Co-expression percentages of PER2 and mCherry are indicated for the sgLacZ or CSAC-*Pers* group. (**h**) Co-expression percentages of BMAL1 and mCherry are indicated for the sgLacZ or CSAC-*Bmal1* group. Error bar = standard deviation (n = 1 mouse for sgLacZ; 3–4 mice for CSAC-*Crys*, CSAC-*Pers*, and CSAC-*Bmal1*, respectively).

To demonstrate the utility of CSAC in vivo, we injected CSAC-*Cry*s, CSAC-*Per*s, CSAC-*Bmal1*, or control AAV into the SCN of mice constitutively expressing Cas9 (Fig. 4a). AAV delivery was histologically verified through confocal microscopic analysis of brain sections containing the SCN (Fig. 4b). For the following analysis, only mice with viral infection sites covering the SCN were included (Fig. S3). Thereafter, we performed immunohistochemistry to analyze the protein expression levels of target genes (Fig. 4c–e). The intensity of the anti-CRY2 signal notably decreased in the SCN of CSAC-*Crys*-injected Cas9 KI mice compared to that of sgLacZ-expressed mice (Fig. 4c). Similarly, the anti-PER2 signal and anti-BMAL1 signal decreased in the CSAC-*Pers* (Fig. 4d) and CSAC-*Bmal1* groups (Fig. 4e), respectively. Quantification of the co-expression of the target gene product and mCherry, a marker of CSAC expression, further revealing a consistent reduction in the immunoreactivity of the CSAC-targeted gene product (Fig. 4f–h).

**Figure 5:**
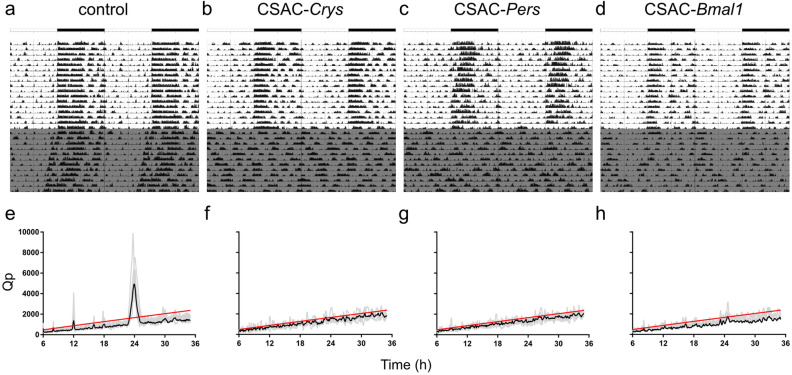
Circadian locomotor activity in SCN-targeted mice. (**a**–**d**) Representative locomotor pattern displayed as a double plot of activity over 28 d in the control (**a**), CSAC-*Crys*- (**b**), CSAC-*Pers*- (**c**), and CSAC-*Bmal1*- (**d**) injected mice. Gray indicates periods of constant darkness. (**e**–**h**) Chi-square periodogram of locomotor activity during constant darkness in control (**e**), CSAC-*Crys*- (**f**), CSAC-*Pers*- (**g**), and CSAC-*Bmal1*- (**h**) injected mice. Gray indicates the periodogram of an individual mouse. The black line indicates the average spectrogram of each group. The red line indicates the significance threshold (*n* = 4–8 mice per group).

To determine the effect of CSAC on the behavioral and physiological aspects of circadian rhythms in awake behaving animals, we used a telemetry-based method to simultaneously record locomotor activity and body temperature. Circadian locomotor activity was normal in control animals injected with AAV expressing fluorescent protein (mCherry; Fig. 5a), which exhibited light-suppressed locomotion during the light phase and a distinct behavioral onset, which aligned well with the point at which the light was turned off. Under constant darkness (DD), the control animals clearly displayed a circadian wake and resting cycle with a period slightly shorter than 24 h, characteristic of the C57BL/6J strain^34^. However, the mice injected with CSAC-*Cry*s or CSAC-*Per*s displayed arrhythmicity under DD (Fig. 5b,c). These mice were subjected to a 12:12-h light/dark (LD) cycle to maintain a daily pattern of locomotor activity, which was completely mitigated under DD. In the CSAC-*Bmal1-*injected group, circadian locomotor activity was largely obliterated during DD and generally displayed lower locomotor activity during LD than the other groups (Fig. 5d). Chi-square periodogram analyses clearly indicated that CSAC disrupted circadian locomotor activity^35^. In the control animals, the average Qp value significantly peaked slightly before 24 h (Fig. 5e), typical of normal circadian locomotor activity, whereas the average Qp values of CSAC-*Crys*, CSAC-*Pers*, and CSAC-*Bmal1* groups were not significant at any time point (Fig. 5f–h).

**Figure 6:**
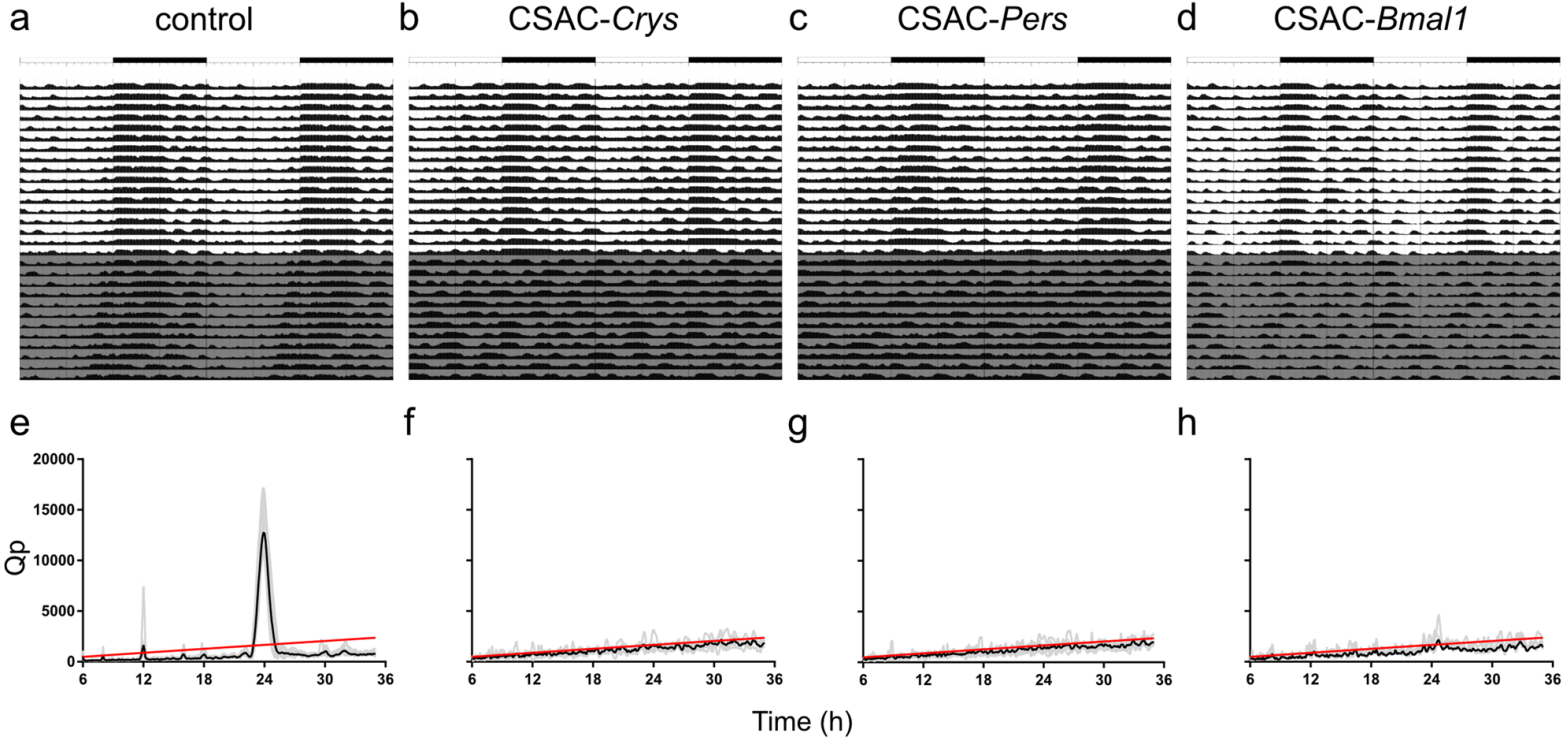
Circadian body temperature of SCN-targeted mice. (**a**–**d**) Representative body temperature profile shown as a double plot over 28 d for control (**a**), CSAC-*Crys*- (**b**), CSAC-*Pers*- (**c**), and CSAC-*Bmal1*- (**d**) injected mice. Gray indicates periods of constant darkness. (**e**–**h**) Chi-square periodogram of body temperature during constant darkness in control (**e**), CSAC-*Crys*- (**f**), CSAC-*Pers*- (**g**), and CSAC-*Bmal1*- (**h**) injected mice. Gray line indicates the periodogram of an individual mouse. Black line indicates the averaged spectrogram of each group. Red line indicates the significance threshold (*n* = 4–8 mice per group).

Body temperature largely followed the same pattern as locomotor activity in the experimental animals. For instance, control animals displayed a typical circadian actogram under both LD and DD conditions (Fig. 6a), whereas the CSAC-*Cry*s, CSAC-*Per*s, and CSAC-*Bmal1* groups lacked a circadian change in body temperature under free-running conditions without a light cue (Fig. 6b–d). Herein, Chi-square periodograms supported these findings, with the average Qp value of the control animals exceeding the significance threshold at approximately 24 h, unlike the mice in the CSAC-*Crys*, CSAC-*Pers*, or CSAC-*Bmal1* groups (Fig. 6e–h).

**Figure 7:**
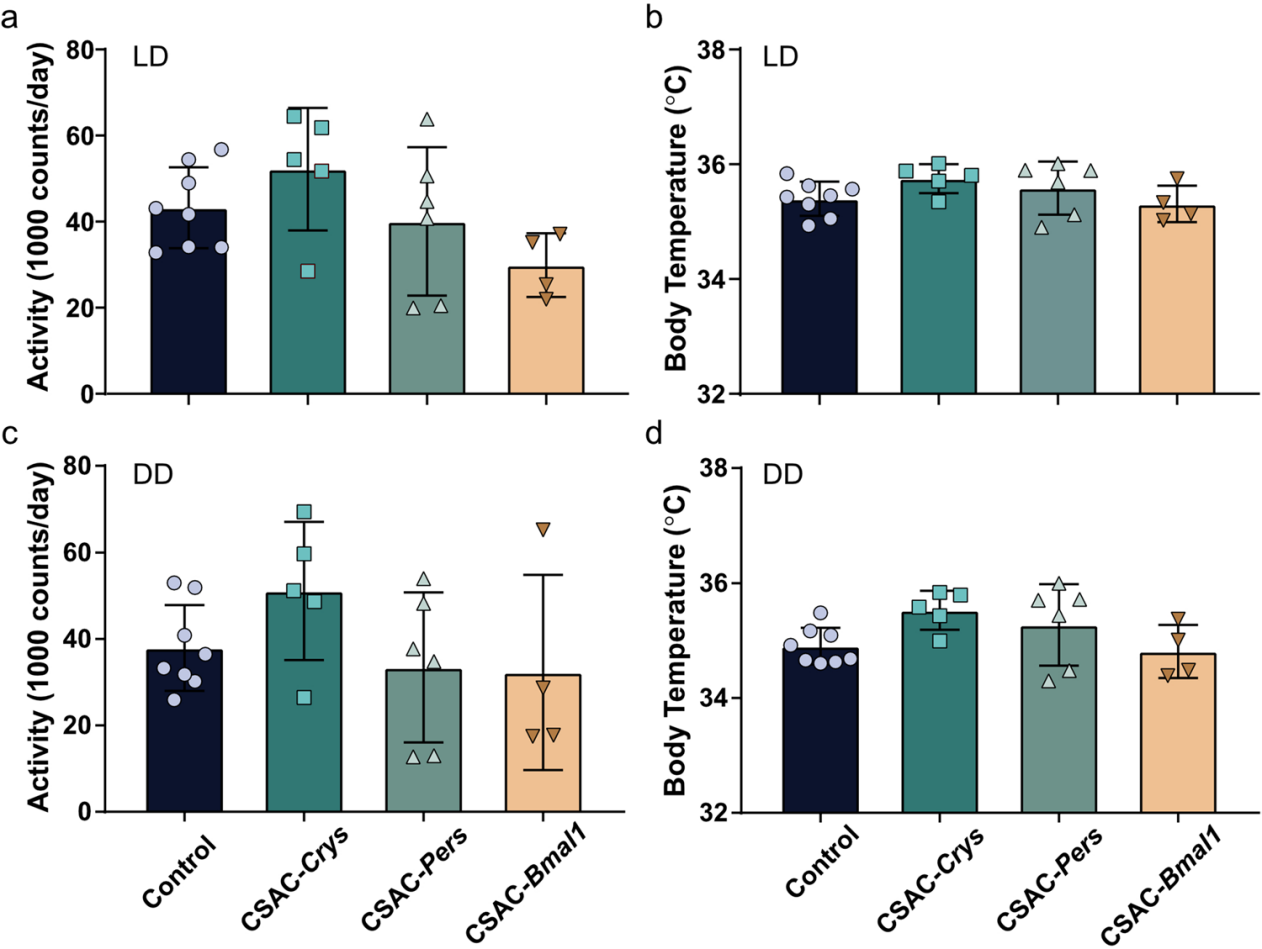
Average locomotor activity and body temperature. (**a**) Average locomotor activity under a 12:12-h light/dark (LD) cycle. (**b**) Average body temperature under an LD cycle. (**c**) Average locomotor activity under constant darkness (DD). (**d**) Average body temperature under DD. Error bar = standard deviation (*n* = 4–8 mice per group).

To determine whether CSAC-mediated depletion of circadian proteins in the SCN affected baseline activity levels or temperature, we determined the average activity (Fig. 7a,c) and body temperature (Fig. 7b,d), respectively. Average locomotor activity and body temperature did not significantly differ between the control and CSAC groups. Together, these findings indicate that CSAC injection into the SCN efficiently and specifically suppressed the physiological hallmarks of the circadian rhythm in vivo without affecting average locomotor activity or body temperature.

## Discussion

In this study, we devised CSAC, a system of multiplexed sgRNAs targeting redundant core clock components packaged in a single AAV, to mitigate molecular clock oscillation by inducing null mutations into several core clock genes. We selected the multiplexed sgRNAs on the basis of their mutation-introducing efficacy and target loci to maximize the possibility of null mutations in the target genes. By targeting core clock genes (*Cry*s, *Per*s, or *Bmal1)* using multiplexed sgRNAs, we knocked down target genes in vitro and in vivo. Furthermore, we reported the value of CSAC in multiple systems including cell lines, organotypic tissue cultures, and live animals.

To our knowledge, this study is the first to develop a set of in vivo applicable viral vectors targeting members of core clock gene families employing CRISPR-Cas9-based genome editing, while CRISPR-Cas9-mediated genome editing has already been applied in in vitro systems or in vivo single targets in chronobiology studies. Korge et al. first reported CRISPR-Cas9-based alteration of the genes involved in the circadian rhythm by targeting F-box and leucine-rich repeat protein 3 (*Fbxl3*)^23^. In doing so, they transfected U2-OS cells stably expressing a Bmal1-Luciferase reporter with a lentivirus expressing Cas9 and two sgRNA targeting different sites in the *Fbxl3* gene and reported that CRISPR-Cas9-based *Fbxl3* KO dampened *Bmal1* promoter-driven luminescence oscillation. The same group then utilized a CRISPR-Cas9 system to generate CRY1 and CRY2 double KO cells^36^. Furthermore, *Clock* targeting has been efficient in mouse embryonic stem cells when investigating the non-clock functions of the *Clock* gene^25^, while Lee and colleagues expanded the repertoire of direct CRISPR-Cas9 targets by designing an adenoviral vector system for efficiently generating null mutant cell lines^28^. Similarly, Herzog and colleagues used AAV vectors to target *Bmal1* in astrocyte-specific *Cre*-driven Cas9-expressing mice^29^, thus facilitating the application of genome editing in vivo in adult animals. These studies provided a foundation for the CSAC developed in this study, offering an advanced approach for knocking out the molecular clock in vivo.

Efficient CSAC-mediated KO of core clock components in Cas9-expressing mice indicates that the multiplexed sgRNAs appropriately recognizes target genomic loci. The expanding repertoire of CRISPR-Cas9 technologies suggests that CSAC could be utilized for various applications beyond the KO of target genes^37^. In this study, we used *Streptococcus pyrogen* Cas9 (SpCas9), which contains two nuclease domains, RuvC and HNH. Mutations in these two domains (Cas9 D10A H840A) can lead to a non-catalytic Cas9 mutant (dCas9) that can bind to target genomic loci as dictated by sgRNA, but cannot generate a DSB^38^. The conjugation of dCas9 to either a transcriptional activator (dCas9-VP64 and dCas9-p65) or repressor (dCas9-KRAB) efficiently induces or suppresses specific sgRNA-target genes^39–41^; thus, intra-coding sequence-targeting sgRNAs such as CSAC are potentially applicable gene regulators. When combined with the conditional expression of dCas9 derivatives using chemical or optogenetic approaches^37^, CSAC may temporally regulate core clock genes to mimic the circadian regulation of gene expression. Furthermore, Cas9 variants targeting RNA could be utilized to visually track *Pers* or *Crys* mRNA in live cells^42^. Considering the increasing molecular repertoire of CRISPR-Cas9 technologies, we believe that the applications of CSAC would expand accordingly.

In conclusion, this study describes the development and validation of CSAC, a powerful and accessible tool for ablating the molecular clock, which can efficiently downregulate core clock genes, dampen their molecular oscillation ex vivo, and disrupt physiological circadian outputs, such as locomotor activity and body temperature. Together with a Cre-dependent Cas9 expression system and Cre driver line, CSAC offers a potent and practical tool to investigate the role of the molecular clock in specific cell types of interest and its applications may potentially expand alongside advancements in CRISPR-Cas9-based genome editing.

## Methods

### Animals

All procedures were approved by the Institutional Animal Care and Use Committee of Daegu Gyeongbuk Institute of Science and Technology (DGIST). All methods were carried out in accordance with the approved animal procedures and laboratory safety guidelines of DGIST. C57BL/6J mice were born and reared in standard mouse cages (16 × 36 × 12.5 cm^3^) with food and water available ad libitum. Mice were weaned at 3–4 weeks of age and housed together with sex-matched siblings with up to four animals per cage. Mice were maintained at a 12:12-h light/dark cycle at 22 ± 1 °C. The number of total animals used in the study is 46 (11 for ex vivo slice preparation; 12 for histological analysis; 23 for circadian measurement of locomotor activity and body temperature). We selected a small sample size as possible because the study aimed to demonstrate the reliability and effectiveness of CSAC in the small sample size. PER2::LUC knock-in mice were generously provided by Joseph Takahashi^31^. Cas9-expressing mice were provided by Feng Zhang^32^.

### Cell culture and mutation detection

Cells were cultured using a previously described method with minor modifications^43^. Neuro2a and Neuro2a Cas9-GFP-Hygor^res^(CSC-RO0033, Genecopeoia, Rockville, MD, USA) cells were cultured in Dulbecco’s modified Eagle medium (DMEM) (Hyclone, Chicago, IL, USA) containing 1 × penicillin/streptomycin (Capricorn Scientific, Ebsdorfergrund, Germany), 10 mg/mL hygromycin (H-34274, Merck Millipore, Burlington, MA, USA), and 10% fetal bovine serum (FBS) (Hyclone) at 37 °C in 5% CO_2_. Neuro2a-Cas9 cells were transfected with pAAV-U6-sgBMAL-hSyn-mCherry, pAAV-U6-sgCRY-hSyn-mCherry, pAAV-U6-sgPER-hSyn-mCherry, or pAAV-U6-sgLacZ-hSyn-mCherry using Lipofectamine 2000 transfection reagent (#11668019, Thermo Scientific, Waltham, MA, USA), in accordance with the manufacturer’s instructions. Cells were harvested after 48 h and genomic DNA was extracted using a tissue mini kit in accordance with the manufacturer’s instructions (Cosmo Genetech, Seoul, South Korea). Surveyor mutation detection assays were performed in accordance with the manufacturer’s instructions (Integrated DNA Technologies, Coralville, IA, USA).

For Sanger sequencing-based analysis of targeted sites, Neuro2a cells were transfected with pSpCas9(BB)-2A-GFP, pAAV-U6-sgBMAL-hSyn-mCherry, pAAV-U6-sgCRY-hSyn-mCherry, pAAV-U6-sgPER-hSyn-mCherry, or pAAV-U6-sgLacZ-hSyn-mCherry, using the TransIT-X2 Dynamic Delivery System (Mirus Bio, Madison, WI, USA, MIR6005) in accordance with the manufacturer’s instructions. Cells were sorted after 48 ~ 72 h, using a BD FACSAria III Cell Sorter (Becton, Dickinson and Company, Franklin Lakes, NJ, USA). Genomic DNA was extracted using a tissue mini kit in accordance with the manufacturer’s instructions (Cosmo Genetech, Seoul, South Korea, CME0112). PCR was conducted to amplify the target genomic locus with a set of primers (Fig. S4v). Amplicons were purified using the QIAquick PCR Purification Kit (28104, QIAGEN, Hilden, Germany) in accordance with the manufacturer’s instructions. PCR amplicons were cloned into pGEM-T Easy Vector (Promega, Madison, WI, USA, A1360). Inserts were sequenced through Sanger sequencing (Macrogen, Seoul, South Korea).

### Plasmids and AAV production

DNA oligomers of sgRNAs designed using CHOPCHOP^30^ with adaptors for cloning were commercially synthesized by Bionics (Seoul, South Korea). After annealing, the DNA fragments containing sgRNAs were cloned into the sgRNA expression vector, pAAV-hU6-gRNA-hSyn-mCherry-KASH. The U6-sgRNA expression cassettes were multiplexed using the Goldengate assembly method, yielding three multiplexed cassettes assembled in pAAV-(hU6-gRNA)X3-hSyn-mCherry-KASH. The plasmid DNA sequences were validated through Sanger sequencing (Macrogen, Seoul, South Korea). AAVs were produced using the triple transfection method and purified using the iodixanol gradient method, as previously described^44^. All AAV vectors administered herein were tittered between 10^12^ and 10^13^ viral genome copies per milliliter (GC/mL), as quantified through qPCR: AAV-DJ-hU6-sgLacZ-hSyn-mCherry-KASH, 1.8 × 10^13^ GC/mL; AAV-DJ-CSAC-Crys-hSyn-mCherry-KASH, 1.1 × 10^13^ GC/mL; AAV-DJ-CSAC-Pers-hSyn-mCherry-KASH, 3.1 × 10^13^ GC/mL; AAV-DJ-CSAC-sgBmal1-hSyn-mCherry-KASH, 6.6 × 10^12^ GC/mL; AAV-2-hSyn-mCherry, 4.7 × 10^12^ GC/mL.

### Western blot analysis

Cell were washed twice with phosphate-buffered saline and lysed through sonication in radioimmunoprecipitation assay (RIPA) buffer (#9806, Cell Signaling, Danvers, MA, USA) and then incubated on ice for 30 min. Thereafter, lysates were centrifuged and the supernatants were boiled for 5 min in Laemmli sample buffer (#NP0007, Invitrogen, Waltham, MA, USA). Proteins were separated on 4–15% Tris–glycine (TG) gels (#45101080003-2, SMOBIO, Hsinchu, Taiwan) and electro-transferred onto polyvinylidene fluoride (PVDF) membranes (#A16646282, GE Healthcare, Chicago, IL, USA) in TG buffer. The membranes were blocked in Tris buffered with Tween-20 (TTBS) with 1% bovine serum albumin (BSA) and incubated overnight at 4 °C in TTBS with 0.1% BSA with the following primary antibodies: anti-mPer1 (#AB2201, Merck Millipore), anti-PER2 (#AB2202, Merck Millipore), anti-BMAL1 (#NB100-2288, Novus, Centennial, CO, USA), anti-CRY1 (#AF3764-SP, R&D Systems, Minneapolis, MN, USA), anti-CRY2 (#HPA037577, Atlas Antibodies, Bromma, Sweden), and anti-β-actin-HRP (#SC-47778, Santa Cruz, Dallas, TX, USA) antibodies. Anti-Rabbit (SKU#31460, Thermo Scientific) or anti-goat (Jackson Laboratory, Bar Harbor, ME, USA) antibody was used as the secondary antibody. Protein bands were detected using enhanced chemiluminescence (ECL) Select Solution (GE healthcare) and an ECL Pico System (ECL-PS100, Dongin, Seoul, Korea).

### Bioluminescence monitoring of organotypic SCN slice cultures

SCN explant cultures were prepared and monitored using a previously described method with minor modifications^45^. One-week-old mPer2::LUC;Cas9 mice were euthanized and their brains were quickly dissected out, chilled on ice, and moistened in Gey’s Balanced Salt Solution (GBSS) supplemented with 0.01 M HEPES and 36 mM D-glucose, and aerated with 5% CO_2_ and 95% O_2_. The brains were then coronally cut into 400-µm-thick slices, using a Leica VT1000 S vibratome (Leica, Wetzlar, Germany). The slices were maintained on a culture insert membrane (Millicell-CM; Millipore, Bedford, MA, USA) and dipped in culture medium (50% minimum essential medium, 25% GBSS, 25% horse serum, 36 mM glucose, and 1 × antibiotic–antimycotic) at 37 °C. The SCN slices were cultured for > 2 weeks before being used in experiments. To quantify bioluminescence, the SCN cultures were maintained in a 35-mm petri dish with 1 mL culture medium containing 0.3 mM D-luciferin (Promega, Madison, WI, USA) at 36 °C. Light emission was measured and integrated for 1 min at 10-min intervals using a dish-type wheeled luminometer (AB-2550 Kronos-Dio; ATTO, Tokyo, Japan). Unless otherwise specified, all culture media and supplements for organotypic slice culture were purchased from Thermo-Fisher Scientific.

SCN slices were virally transduced after 7 d of stable culturing, as described previously^46^. AAVs were dropped directly onto the surface of each SCN slice (1–2 μL per slice) in a 35-mm dish and incubated for 15 d before bioluminescence monitoring.

### Stereotaxic injection

Surgery was aseptically carried out. Briefly, mice were anesthetized through intraperitoneal injection of a mixture of ketamine (100 mg/kg) and xylazine (10 mg/kg) and placed in a stereotaxic apparatus (RWD Life Science, Shenzhen, China), where their brains were injected with AAVs (bilateral injection, 250 nL each) using a Nanoliter injector system (WPI, Sarasota, FL, USA) at a rate of < 0.1 μL/min in the SCN (AP = − 0.8 mm, ML =  ± 0.22 mm from the bregma, DV = − 5.85 mm from the brain surface).

### Measurement of locomotor activity and body temperature

Locomotor activity and body temperature of the mice were measured using E-mitter, a radio transmitter-based telemetry system (Starr Life Science, Oakmont, PA, USA). E-mitter was aseptically implanted beneath the skin on the dorsa of the mice under general anesthesia induced through intraperitoneally administered ketamine (100 mg/kg) and xylazine (10 mg/kg)^47^. After implantation, the mice were allowed to recover for at least 1 week and acclimatized at a regular 12:12-h light/dark cycle. Activity and temperature data detected using the implanted sensor were transmitted to a receiver (ER-4000 engergizer receiver, Starr Life Science). The allocation of the mice was counterbalanced and randomized in the experimental batch. We also randomized and counterbalanced the location of the test cages. Data acquisition and digital transformation were performed using VitalView software every 6 min (Starr Life Science, https://www.starrlifesciences.com/product/activity-software/). Automated computation pipeline provided unbiased data analysis of circadian phenotypes.

### Immunohistochemistry and confocal microscopy

After allowing for the expression of virally transduced genes for 3 weeks, mice were intracardially perfused with 4% paraformaldehyde and post-fixed at 4 °C overnight. To inspect the injection sites, coronal sections (50-μm thick) were obtained using a Leica VT1000 S vibratome (Leica, Wetzlar, Germany). Through visual inspection, we drew an outline enclosing the transduced area on the fluorescent image containing the most number of transduced cells for each animal. For immunohistochemical analyses, The brains were immersed in 30% sucrose in PBS and dehydrated for 3 d at 4 °C. The brains were embedded in optimal cutting temperature (OCT) compound (Scigen, Paramount, CA, USA) and frozen at − 80 °C. Coronal sections (20-μm-thick) were obtained using a Leica CM3050 S cryostat (Leica). The prepared slices were labeled using the following primary antibodies: rabbit anti-CRY2 (Atlas antibodies, Stockholm, Sweden, HPA037577, 1:50), rabbit anti-PER2 (Millipore, Burlington, MA, USA, AB2202, 1:1000), and rabbit anti-BMAL1 (Novus, Saint Charles, MO, USA, NB100-2288, 1:1000). The following fluorophore-conjugated secondary antibodies were used: Alexa 647 donkey anti-rabbit (Invitrogen, Waltham, MA, USA, A31573, 1:200). The nucleus was counterstained with 4′6′-diamidino-2-phenylindole (DAPI) and images were captured using a Nikon C2^+^ confocal microscope system (Nikon, Tokyo, Japan) and linearly adjusted with Fiji^48^.

### Data and statistical analysis

Chi-square periodogram analysis was performed using the xsp package^49^ in R^50^. Real-time bioluminescence was analyzed using the cosinor procedure to determine the amplitude, period, and robustness value for each bioluminescence trace^51,52^. Graphs were plotted constructed using Prism 8 (GraphPad, SanDiego, CA, USA) or ggplot2^53^. The threshold for statistical significance was set at *P* < 0.05. The study was carried out and reported in compliance with the ARRIVE guidelines 2.0^54^.

## Supplementary Information

Supplementary Information

## Data Availability

All data generated and analysed during this study are included in this article.
