# Supplementary material for: Multiplexed CRISPR-Cas9 system in a single adeno-associated virus to simultaneously knock out redundant clock genes
Source: Sci Rep. 2021 Jan 28;11:2575. doi: 10.1038/s41598-021-82287-0 (PMC7844015; doi:10.1038/s41598-021-82287-0)
Supplement: Supplementary file 1 — Supplementary Information [file 41598_2021_82287_MOESM1_ESM.pdf]

Supplementary material

Multiplexed CRISPR-Cas9 system in a single adeno-associated virus to simultaneously knock out redundant clock genes

Boil Kim[1], Jihoon Kim[1], Minjeong Chun[1], Inah Park[1], Damhyeon Kwak[1], Mijung Choi[1], Kyungjin Kim[1], Han Kyoung Choe[1, 2, 3*]

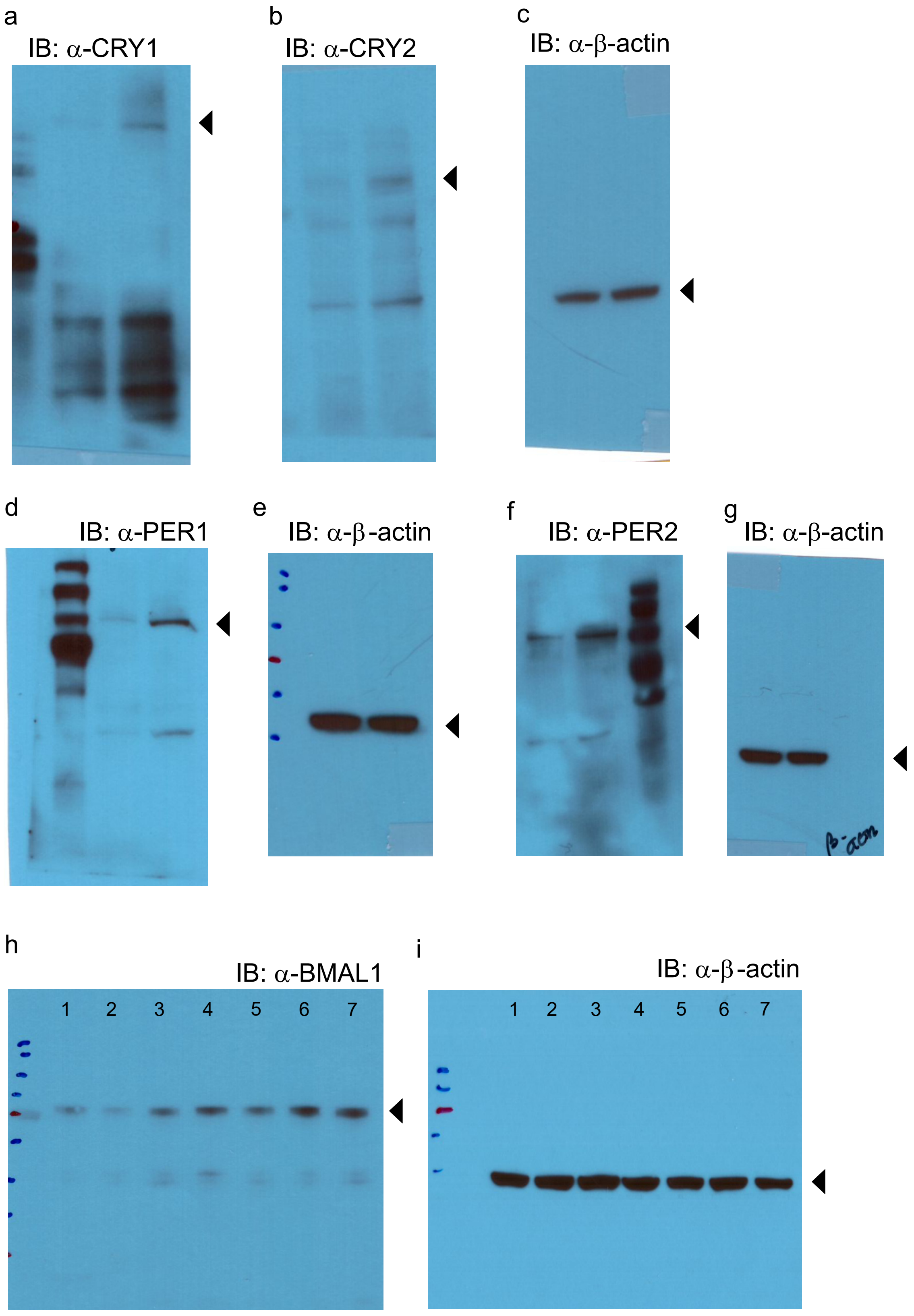

Figure S1. Full-length western blots shown in Figure 2. Representative western blots of protein extracts from Neuro2a-Cas9 cells transfected with CSAC-*Crys* (a-c), CSAC-*Pers* (d-g), or CSAC-*Bmal1* (h, i). Each blots were probed with the antibodies shown above. For h and i, lane 2 and 3 were cropped and shown in Figure 2, while other lanes are irrelevant to the current work. Filled arrows indicate the positions of detected proteins.

a

sgCry1#2

```
CCGCTG-CGTCTATATCCTCGACC
14bp-------GATATCCTCGACC
5bp-----------ATCCTCGACC
CCGCTG----TTATATCCTCGACC
CCGCTG----TTATATCCTCGACC
CCGCT---GTCTATATCCTCGACC
CCGCT---GTCTATATCCTCGACC
CCGCT--CGTCTATATCCTCGACC
CCGCTGCCGTCTATATCCTCGACC
CCGCTGCCGTCTATATCCTCGACC
CCGCTGCCGTCTATATCCTCGACC
CCGCTGCCGTCTATATCCTCGACC
CCGCTGCCGTCTATATCCTCGACC
CCGCTG-CGTCTATATCCTCGACC
```

b

sgCry2#1,2

```
CCTCGT-CTGTGGGCATCAACCGA-TGGAGG
CCTCGT-CTGTGGGCATCAACCGA-TGGAGG
CC-----------------------TGGAGG
6bp----CTGTGGGCATCAACCGA--GGAGG
CCACG---TGTGGGCATCAACC-----GAGG
CCTCG--CTGTGGGCATCAACC------AGG
CCTCGT-CTGTGGGCGTC-----A-TGGAGG
C------CTGTGGGCATCAACCGA-TGGAGG
CCTCG---TGTGGGCATCAAC----TGGAGG
CCTCGTCCTGTGGGCATCA----A-TGGAGG
CCTCTG-CTGTGGGCATCAACCGATTGGAGG
CCTCGT-CTGTGGGCATCAACCGA-TGGAGG
CCTCGT-CTGTGGGCATCAACCGA-TGGAGG
CCTCGT-CTGTGGGCATCAACCGA-TGGAGG
CCTCGT-CTGTGGGCATCAACCGA-TGGAGG
```

c

sgPer1#3

```
CCTACT-CATTGCCGAGCGCATCC
7bp---------------GCATCC
----------------------CC
C---------TGCCGAGCGCATCC
CC-------TTGCCGAGCGCATCC
C------CATTGCCGAGCGCATCC
CCTAC----TTGCCGAGCGCATCC
C---CT-CATTGCCGAGCGCATCC
CCTAC----TTGCCGAGCGCATCC
CCTACT---TTGCCGAGCGCATCC
CCTAC--CATTGCCGAGCGCATCC
CCTACTGCATTGCCGAGCGCATCC
CCTACT-CATTGCCGAGTGCATCC
```

d

sgPer2#1

```
CCGGCCCTTGATGCTCGCCATCC
CCGGCC--TGATGCTCGCCATCC
CCGGCC--TGATGCTCGCCATCC
CCGG-CCTTGATGCTCGCCATCC
CCGG-CCTTGATGCTCGCCATCC
CCGG-CCTTGATGCTCGCCATCC
CCGG-CCTTGATGCTCGCCATCC
CCGG-CCTTGATGCTCGCCATCC
CCGGCCCTTGATGCTCGCCACCC
CCGGCCCTTGATGCTCGCCATTC
CCGGCCCTTGATGCTCGCCATCC
CCGGCCCTTGATGCTCGCCATCC
CCGGCCCTTGATGCTCGCCATCC
```

e

sgPer2#2

```
GAGCACAACCCCTCCA-CGAGCGG
GAGCAC-------------AGCGG
GAG-----------CA-CGAGCGG
GAGCACAACCCCTCCA------GG
GAGCACAA-----CCA-CGAGCGG
GAGCACAACCCCT----CGAGCGG
GAGCACAACCCCT----CGAGCGG
GAGCACTACCCCTC---CGAGCGG
GAGCACAACCCCTCCA-C--GCGG
GAGCACAACCCCTCC--CGAGCGG
GAGCACAACCCCTCCA-C-AGCGG
GAGCACAACCCCTCCACCGAGCGG
GAGCACAACCCCTCCACCGAGCGG
GAGCACAACCCCTCCACCGAGCGG
GAGCACAACCCCTCCACCGAGCGG
GAGCACAACCCCTCCACCGAGCGG
GAGCACAACCCCTCCA-CGAGCGG
GAGCACAACCCCTCCA-CGAGCGG
```

f

sgBmal1#1

```
CCACCG-ACCTACTCTCCGGTTCC
--------------------CCCC
6pb--G-CCCCACTCTCCGGTTCC
CCA------CTACTCTCCGGTTCC
CCACCG-A-----TCTCCGGTTCC
CCA------CTACTCTCCGGTTCC
CCACCG-----ACTCTCCGGTTCC
CCACCG---TTACTCTCCGGTTCC
CCACCG---CTACTCTCCGGTTCC
CCACCG-A-CTACTCTCCGGTTCC
CCACCG--CCTACTCTCCGGTTCC
CCACTGAACCTACTCTCCGGTTCC
CCACTGAACCTACTCTCCGGTTCC
CCACCGAACCTACTCTCCGGTTCC
CCACCGAACCTACTCTCCGGTTCC
CCACCG-ACCTACTCTCCGGTTCC
CCACCG-ACCTACTCTCCGGTTCC
CCACCG-ACCTACTCTCCGGTTCC
CCACCG-ACCTACTCTCCGGTTCC
```

g

sgBmal1#3

```
CCACAGTCAGATTGAAAAGAGGC
14bp----------------6bp
CCACA--------GAAAAGAGGC
CCA----CAGATTGAAAAGAGGC
CCA----CAGATTGAAAAGAGGC
CCACAGTCAGATTGAAAAGAGGC
CCACAGTCAGATTGAAAAGAGGC
CCACAGTCAGATTGAAAAGAGGC
CCACAGTCAGATTGAAAAGAGGC
CCACAGTCAGATTGAAAAGAGGC
CCACAGTCAGATTGAAAAGAGGC
CCACAGTCAGATTGAAAAGAGGC
CCACAGTCAGATTGAAAAGAGGC
CCACAGTCAGATTGAAAAGAGGC
```

Figure S2. Sanger sequencing of on-target sites. (a, b) Sequencing results of CSAC-*Crys*-transfected Neuro2a cells. Target site of sgCry1#2 (**a**), sgCry2#1, 2 (**b**) are shown. **(c-e)** Sequencing results of CSAC-*Pers*-transfected cells. Target site of sgPer1#3 (**c**), sgPer2#1 (**d**), and sgPer2#2 (**e**) are shown. **(f-g)** Sequencing results CSAC-*Bmal1*-transfected cells. Target site of sgBmal1#1 (**f**) and sgBmal1#3 (**g**) is shown. Blue or light blue sequences indicate wildtype sequences with PAM sequence (orange). The sequences of each analyzed clone is presented in following columns in black.

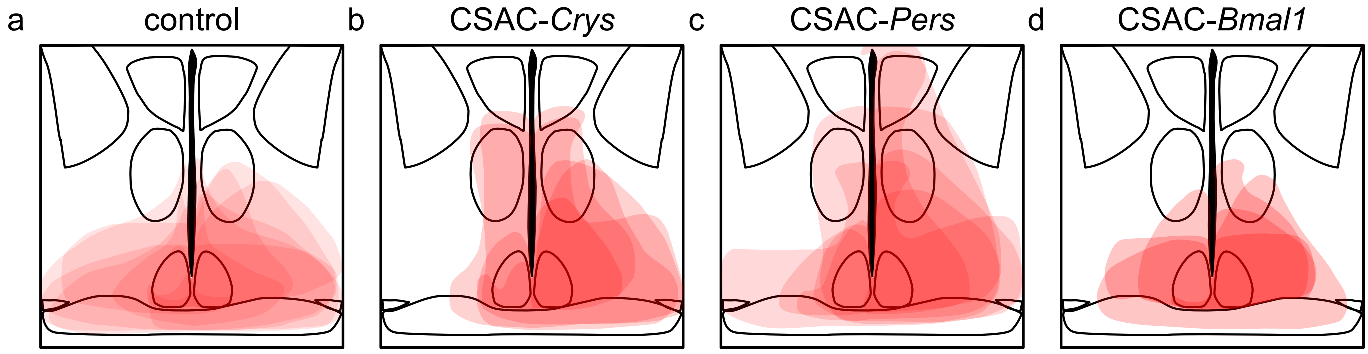

Figure S3. Injection sites of CSAC-injected mice. Injection site of control virus (**a**), CSAC-*Crys* (**b**), CSAC-*Pers* (**c**), and CSAC-*Bmal1* (**d**). Light red indicates the infected areas of each animal.

Primer name		Sequence (5'→3')	Target size (bp)
SUR sgBmal1#1	FWD	TGTGATGCTTCTCCCAGTCC	756
	REV	GGCCCGGCTTTAAGGCTATT	
SUR sgBmal1#3	FWD	TTGGAAGCAGTCACAACTCA	760
	REV	GATCATTGCAAGGGGTGGGA	
SUR sgCry1#2	FWD	GAAGGTATGCGTGTCCTCCG	884
	REV	CCTTGCTTTTCCTCCTCTAACCT	
SUR sgCry2#1	FWD	CTGGTTCCGCAAAGGACTAC	484
	REV	GTGGGGGAGAGTCAAACAAA	
SUR sgCry2#2	FWD	GAGGCGATCCAGACTCCTTG	587
	REV	CACACCCCTAAAAGCCCAGT	
SUR sgPer1#3	FWD	GCTCTCATGTTGTGGGCTCT	573
	REV	GCTCAGGGACCTCATCCAAC	
SUR sgPer2#1	FWD	CTGACTGCCTTGTGTCCCTT	397
	REV	TCCTGTGCTGTCTACTCCCA	
SUR sgPer2#2	FWD	CCCTGGTCAGTTTTATCGGCT	351
	REV	ACTCAGAAGTACAGTGCCCG	
sgBmal1#1	FWD	CAGCGACTTCATGTCTCCGGGC	175
	REV	TGACAGCTATGGGCAGGCAGGT	
sgBmal1#3	FWD	CGTTGTTTCGATGCTCGTGT	200
	REV	TGTGCTGAACAGCCATCCTT	
sgCry1#2	FWD	TGCACTGGTTCCGAAAGGG	208
	REV	CTAACGCATCGACACCCGA	
sgCry2#1,2	FWD	CTGCGTCTACATCCTCGACC	159
	REV	GGCAAAAACGCTGTGTCAGA	
sgPer1#3	FWD	TACCAGCCATTCCGCCTAAC	175
	REV	ACCCCATCCTAGAGGGAACC	
sgPer2#1	FWD	TCCCCCTCCTGGGCTATCTA	135
	REV	CACAGGCCTTGGGGCATAAA	
sgPer2#2	FWD	TCCCTGGTCAGTTTTATCGGC	200
	REV	GGTGGGCTCCACATCACTAA	
sgBmal1#1 off1	FWD	TGCATTCTCATTTTCCCCCTCTTCCA	235
	REV	CTCACTCCCAGGAGCAGCTGGT	
sgBmal1#3 off1	FWD	CTGGATGTGGCATGGCTGCTGA	198
	REV	TCCTCCAAACACAGGCCTCCCT	
sgBmal1#3 off2	FWD	GAAGCGGCCTTAGGCTTGGCTC	326
	REV	TGCTCATTGGGCTCTGGTGGGA	
sgBmal1#3 off3	FWD	AAAACACTTGGCCTGGCCTCCC	201
	REV	GGGGGAAGTGTGTGTCCAGACC	
sgCry1#2 off1	FWD	CTGGAGTCCATCTACTTGAGCT	588
	REV	TCATGTCTGTCCTCAAGCTGTA	
sgCry2#1 off1	FWD	CTCCCCGATCCGTGCAGTGGTA	311
	REV	TGGCCTGCCTCACTTACCCTCC	
sgCry2#1 off2	FWD	CTGTGTCAGCTTCTGCCCAGCC	153
	REV	GTCACTCGGCAGCACCCAGAAC	
sgCry2#2 off1	FWD	ACCTGGGCTGAGCTTGGGTCTT	340
	REV	TGTGACATGTAGCGTGGGAAAGCC	
sgCry2#2 off2	FWD	AGCACTAGGGGGCAAGGAGACC	182
	REV	AGCCCAGGACCACCGCTAATGT	
sgCry2#2 off3	FWD	GCGGTAGGCATGTCTGTGGGGA	326
	REV	TGGGGGAGGGTGGTAATGGTTGA	
sgPer1#3 off1	FWD	GATCACGTTGCCTCCAGCTGCA	316
	REV	AGGTCCTGGGTGGTGACATGCA	
sgPer2#1 off1	FWD	CAGTACACGGGGCTGGGATGGA	221
	REV	AGCCACCGTTGAGAGGGGAGTC	
sgPer2#1 off2	FWD	AGCCACCGCTGGAGTTTTGTGG	331
	REV	CAGGCCACAGCGTTGGAGACAG	
sgPer2#1 off3	FWD	GGTCCGAGTGCTGGTTCTGTGC	107
	REV	CAGCCTCCAAGCACGAGAACCG	
sgPer2#2 off1	FWD	GGTGAACTGGGAATGGTGTGGGC	195
	REV	ATTTGGGGTGGGGGTTAGGGGG	

Figure S4. Primer information.
